# *ChroMo*, an Application for Unsupervised Analysis of Chromosome Movements in Meiosis

**DOI:** 10.3390/cells10082013

**Published:** 2021-08-06

**Authors:** Daniel León-Periñán, Alfonso Fernández-Álvarez

**Affiliations:** Andalusian Center for Developmental Biology (Pablo de Olavide University/Consejo Superior de Investigaciones Científicas/Junta de Andalucía), 41013 Sevilla, Spain; dleoper@upo.es

**Keywords:** chromosome movements, meiosis, data mining, web platforms, fission yeast

## Abstract

Nuclear movements during meiotic prophase, driven by cytoskeleton forces, are a broadly conserved mechanism in opisthokonts and plants to promote pairing between homologous chromosomes. These forces are transmitted to the chromosomes by specific associations between telomeres and the nuclear envelope during meiotic prophase. Defective chromosome movements (CMs) harm pairing and recombination dynamics between homologues, thereby affecting faithful gametogenesis. For this reason, modelling the behaviour of CMs and their possible microvariations as a result of mutations or physico-chemical stress is important to understand this crucial stage of meiosis. Current developments in high-throughput imaging and image processing are yielding large CM datasets that are suitable for data mining approaches. To facilitate adoption of data mining pipelines, we present *ChroMo*, an interactive, unsupervised cloud application specifically designed for exploring CM datasets from live imaging. *ChroMo* contains a wide selection of algorithms and visualizations for time-series segmentation, motif discovery, and assessment of causality networks. Using *ChroMo* to analyse meiotic CMs in fission yeast, we found previously undiscovered features of CMs and causality relationships between chromosome morphology and trajectory. *ChroMo* will be a useful tool for understanding the behaviour of meiotic CMs in yeast and other model organisms.

## 1. Introduction

Meiosis is an essential process for promoting genetic diversity that allows for the generation of new allelic combinations. After a single round of DNA replication, two consecutive rounds of nuclear division, known as meiosis I (MI) and meiosis II (MII), ensure the correct distribution of chromosomes from diploid parental cells to haploid gametes [1,2,3,4]. The critical stage for safeguarding genetic diversity is the recombination between homologous chromosomes during meiotic prophase. Homologous chromosome search is facilitated by nuclear movements that are driven by cytoskeleton forces [5,6,7]. Actin and dynein are two of the most evolutionary widespread motors for generating forces to move the nucleus [8,9,10,11,12,13,14,15]. Nuclear movements are transmitted to the chromosomes by the formation of the telomere bouquet, a conserved chromosomal configuration in which the telomeres cluster together in a specific region of the nuclear envelope (NE), often close to the centrosome [16,17,18]. Telomere bouquet formation is driven by the expression of the meiotic prophase-specific proteins TERB1 and TERB2 in most metazoans [19,20,21,22,23], HIM-8/ZIM-2/ZIM-1/ZIM-3 in *Caenorhabditis elegans* [24,25], Ndj1 in *Saccharomyces cerevisiae* [26,27], and Bqt1 and Bqt2 in *Schizosaccharomyces pombe* [28]. Once meiotic CMs are complete, the telomere bouquet dissociates from the NE.

Meiotic CMs can take hours, although the duration varies between species, as do the trajectory and morphology of the nucleus and chromosomes [5]. For example, in the fission yeast, they oscillate between a rounded shape and a horsetail shape [29,30]. The formation and disassembly of the telomere bouquet define the two phases of meiotic prophase in fission yeast: (i) the horsetail stage, characterized by the most intense nuclear oscillations; and (ii) the post-horsetail stage, a short segment at meiosis I onset where the telomere bouquet is disassembled without nuclear movement [16,31,32,33,34].

Using tracking schemes to follow the behaviour of CMs, several groups have studied the fundamental patterns of meiotic movement in model organisms such as *C. elegans* [10,35,36,37], *S. cerevisiae* [26,38,39], and *S. pombe* [31,32,33]. These studies often use time-lapse fluorescence microscopy to track a variety of particles ranging from cytoskeletal motor proteins, such as dynein, to specific chromosome loci. To analyse the trajectory of these particles, several approaches are used: (i) mean-squared displacement (MSD), which accounts for the average amount of space that a particle has explored at specific time steps compared to a reference (*zero*) time, is widely used to distinguish between directed, diffusive, sub-diffusive and confined diffusive movements, such as in the case of tracking motor proteins or individual loci [40,41,42,43,44]; (ii) measurements of velocity, usually used to test for differences in the amount of chromosome displacement across experimental groups, i.e., mutants versus wild type (*wt*) [39,45,46]; (iii) analysis of auto- and cross-correlation, to evaluate long-range spatiotemporal patterns [44]; and (iv) spectral approximations, to quantify the frequency of oscillatory-like behaviours [47,48]. In cases where histones or NE proteins are tagged and imaged, their morphology can be determined downstream, allowing differences between groups to be characterized with respect to chromosome or nuclear shape [49,50].

These kinds of techniques, however, have key limitations arising from their time-ensemble nature, as reviewed by Miné-Hattab and Chiolo [51]. For example, different modes of movement may produce the same MSD curves or velocity distributions, as trajectories that are effectively different may nevertheless yield identical distribution summaries [52]. Delimiting smaller time windows could be one way to increase the ability to distinguish behaviours in this situation, so that potentially different modes of diffusion or directed motion can be evaluated [42]. This means that specific patterns of CM, particularly those that are not apparently linked to a known biological process, cannot be easily identified. Recent developments in imaging technologies allow increasingly higher cell counts and higher sampling frequencies per time-lapse experiment; this generates a higher volume of time-series data that could potentially benefit from the application of data mining methods. Ideally, these methods would facilitate an emphasis on the serial nature of data rather than on its distributions. This is because not only the amount of movement but also its trajectory over time can potentially be important for CM as a biological process.

To facilitate the wide adoption of state-of-the-art data mining tools by the CM community, we present the interactive web application *ChroMo* (https://chromo.cloud, accessed on 4 August 2021). Its core technologies consist of well-known tools for unsupervised time-series segmentation and clustering, spectral assessment, motif discovery, and causation network discovery, which can also generate interactive, publication-quality figures. Here, we introduce the key features of the application and highlight its capabilities with synthetic and biological datasets. In this regard, we illustrate how *ChroMo* can be used to study meiotic CMs in fission yeast, whose chromosomes move vigorously and continuously across poles during meiotic prophase for 2–3 h. *ChroMo* will be a useful tool to investigate the nature of CMs in yeast meiosis and other model organisms.

## 2. Materials and Methods

### 2.1. ChroMo Platform

*ChroMo* is a web app developed using Shiny technology. All front-end, back-end, and analysis codes are working under R 4.0.2 (R Core Team, 2020). The ready-to-use version (https://chromo.cloud, accessed on 4 August 2021) is hosted in a DigitalOcean droplet, under ShinyProxy 2.4.0, to allow fine-tuning of concurrency. A Docker image of the *ChroMo* application was built upon the shiny-geospatial image by *cyversevice*, by adding all necessary libraries and source files. The web access is behind a Docker containerized Nginx reverse proxy. Three instances of the same *ChroMo* Docker image were configured, namely S (Small), M (Medium), and L (Large), each one with 256 MB, 512 MB, and 1 GB of RAM, with respectively increasing CPU resource allocation. Apart from the specific time-series analysis tools described in the following sections, the packages *dplyr* 1.0.5, *ggplot2* 3.3.3, *plotly* 4.9.3, and *future* 1.21.0 were used.

### 2.2. Type of Data Required as Input

**Figure 1:**
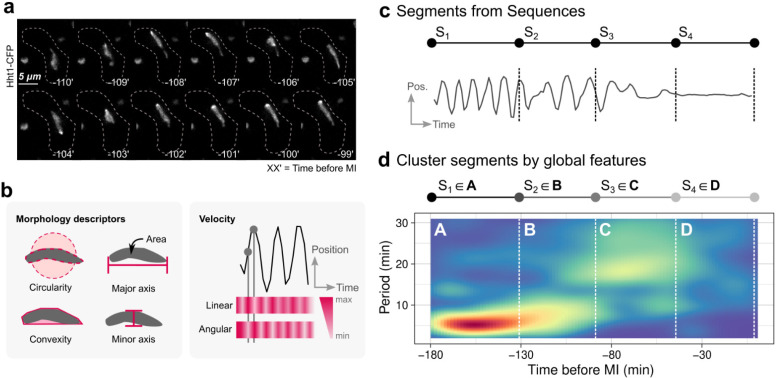
*ChroMo* analysis of chromosome movements in *S. pombe* wild-type settings during meiotic prophase. (**a**) Series of frames of film of a *wt* meiocyte harbouring Hht1-CFP (histone H3 tagged at one of the two endogenous *hht1^+^* loci; chromosomes). Numbering indicates meiotic progression in minutes, t = 0 being just before meiosis I. (**b**) Morphology descriptors—area, circularity, convexity, major and minor axes—as well as linear and angular velocities, are calculated from the chromosome signal of each individually recorded cells. (**c**) An individual example segmentation according to movement of the chromosome mass, described by its angular and linear velocities, is presented. (**d**) Once various individual cells have been segmented, clustering is applied to find relevant groupings of segments that may explain a conserved behaviour. This can be further checked by visualizing the average behaviour of, e.g., the spectrogram (depicted below) of all retrieved sequences standardized in time by the beginning of MI.

The object of study in *ChroMo* are time-series numeric data. Therefore, the user is expected to upload tabular file formats such as XLSX, CSV, or TSV, in which numerical (time-discretized) features are already extracted, e.g., from a dataset of time-lapse images. In this case, the user is responsible for performing adequate image processing and analysis workflows in which feature extraction will lead to the numerical data expected by *ChroMo*. An example of this would be tracking a fluorescent mass of chromosomes over time. First, a segmentation protocol would be needed to distinguish the chromosomal signals from the background, either manually or automatically (see Section 2.9, Fluorescence microscopy, image processing, and analysis). Then, the individual objects from the segmentation need to be tracked between frames to obtain local features for the same object over time. A multitude of characteristics can be extracted from each image, and the choice depends mainly on the biological question. For example, we focus on the study of position and morphology (see Figure 1); in this case, the numerical dataset would be represented in a table with one column per characteristic (time point, Y coordinate, Z coordinate, total area, major axis, etc.).

### 2.3. Synthetic Time-Series Generation

Four behavioural segments were designed to resemble previously chosen time windows from biological data of CM trajectory tracks, as in *wt S. pombe* meiotic prophase. Each segment is composed of a random walk component and a sinusoidal signal with random period and amplitude; decay of oscillations by the reciprocal of time is also used. Time step size was chosen to be 1 min to replicate the sampling settings used in fluorescence live-microscopy. Space is dimensionless, with arbitrary units. Two types of synthetic time-series with four segments, in different ground-truth orderings (see Appendix A.1), were generated using custom code available in *ChroMo*’s repository.

For statistical power assessment, 100 simulations, with varying sample sizes and consisting of 180 time points, were generated for each type; specific use-cases of algorithms and tools in the main figures were carried with a similarly generated dataset consisting of 400 (200 + 200) synthetic tracks.

### 2.4. Time-Series Behavioural Segmentation and Statistics

A preliminary segment-discovery phase, per particle, is executed using one of the two algorithms available; for one- or two-dimensional problems, the package *segclust2d* with segment selection based on Lavielle’s criterion is used [53,54]. Parameters can be specified by the user, with the *ChroMo*-default being *K* = 10 (maximum segments to discover) and *L_min_* = 5 (minimum size of the segment). For higher-dimensional problems, segmentation is performed using the *segmenTier* package [55]. For each calculated segment and per particle, summary statistics and spectral features are calculated for selected covariates. Clustering of segments across all the calculated summary descriptors is performed using finite normal mixture modelling (FNMM) on the *Mclust* package [56]. The minimum and maximum number of clusters to search is, by default, between 1 and 10, and can be user modified. The optimum number of clusters is selected by the segmentation Bayesian Information Criterion (BIC). Then, corresponding clusters are attached as factors to each particle and segment.

Differences in cluster composition are assayed through Binomial Generalized Linear Models (GLMs), i.e., a Logistic Regression model [57]:

(1) $$\Pi_{x}=\frac{e^{\beta_{0}+\sum_{i=1}^{n}\beta_{i}x_{i}}}{1+e^{\beta_{0}+\sum_{i=1}^{n}\beta_{i}x_{i}}}$$

With respect to the binary response variable *Y*, $\Pi_{x}$ is the probability of success, $\Pi_{x}=P\left(Y=1|X=x\right)=1-P\left(Y=0|X=x\right)$, *x_i_* is each of the *n* explanatory variables—and their interactions—and β*_i_* the weights. The *logit* transformation leads to a linear model, $\mathrm{logit}\left(\Pi_{x}\right)=\beta_{0}+\overset{n}{\underset{i=1}{\sum }}\beta_{i}x_{i}$. Following this strategy, we built the following models:

- Interaction of cluster factor, its starting and ending time;
- Interaction of time;
- Interaction of cluster factor and its proportion;
- Interaction of cluster factor;
- Intercept-only.

Analysis of Variance (ANOVA) is used to assess the significance of the interactions against the intercept-only model, with either Chi-Squared (default) or Likelihood Ratio tests. Then, the null hypothesis is the absence of differences in cluster composition (what are the clusters and their relative amount) and/or their duration between groups. For global and segmented time-series, spectral densities, time spectrograms, one and two-dimensional velocity densities, and MSD curves are calculated. In the case of spectral analysis, the significance of different densities is tested with a similar GLM approach. In the case of velocities, the generic non-parametric U-test is used to test for location shift.

Statistical power of the tests above is calculated using our synthetic dataset with the ground truth segmentation mentioned before. Power (1−β) allows one to assess the percentage of cases in which, as expected, a significant result is obtained. We aimed to find (1−β)≥0.8 with *p < 0.05*. This means that the test can reject the null hypothesis when in fact it does not hold in at least 80% of the cases, with a significance level of 0.05.

### 2.5. Global and Per-Segment Motifs Discovery

Motif and discord analyses are performed using the *tsmp* package. MPX and SCRIMP++ algorithms were used to compute the exact or approximate Matrix Profiles [58,59]. By default, a correlation of 0.98 and a number of samples of 1, for the exact case, are used. Window size defaults to 20. All parameters can be further tuned by the user. Unsegmented time-series for a selected variable are concatenated to perform global discovery. In the case of segmented time-series, all segments of belonging to same clusters are concatenated, with the same downstream steps as for the global case. Distribution of motif location is also calculated, per group and segment.

### 2.6. Global Covariate and Time-Series Causality Analysis

The implementation of the PC-algorithm in the *pcalg* library for R is used for causal structure learning, specifically for observational data without hidden variables, and without explicitly considering data as time-series [60,61]. The Variable-Lag approaches for Granger Causality and Transfer Entropy, implemented in the *VLTimeCausality* library for R, are chosen when explicitly treating the data as time-series, with relaxation of the stationary assumption and of the fixed time delay of influence effects [62]. In both cases, for PC-algorithm and VL approaches, a by-default *p*-value of 0.01 is automatically selected and used; also, the adjustment method for multiple testing shall be specified (Bonferroni by default). The adjusted *p*-value is then used for testing the possible causality relations between variables, individually. For each of the tests, an adjacency matrix **A** is individually obtained; each element, *a_ij_*, represents the result of a contrast as the binary value indicating whether a significant relation *i* → (causes) *j* could (1) or not (0) be found. Then, an average-weighted adjacency matrix **Â** is calculated: each position, *â_ij_*, holds the proportion of cases in which the contrast was significant (1). This matrix encodes a directed weighted graph, with direction of an edge being the specific row-column combination for a cell and weight its specific value. A threshold for the minimum percentage needed to consider a relation can be further specified as an input parameter. Interactive and static graph visualisations of the causality network are provided by *igraph*, *networkD3*, and *GGally* packages for R.

### 2.7. Limitations of ChroMo

#### 2.7.1. Which Data Can Be Used?

*ChroMo* eases the analysis of already extracted features, for example, from fluorescence microscopy. Thus, the user must evaluate the quality of data before uploading it to the platform. A common example would be to remove motion artefacts in time-lapse image data before extracting the coordinates of the point; otherwise, the calculations of other descriptors or variables could be incorrect.

#### 2.7.2. Parameterization and Interpretation

Segment discovery is only a data-driven analysis; therefore, it does not provide any explanation as to what the biological significance of the segments is. An additional interpretation must be taken into account to acquire this knowledge, that is, to correlate these discovered segments with other already known events during that same period of time in the cell cycle (DNA replication, homolog recombination, etc.). Furthermore, the minimum size of detectable motifs depends on the number of clusters; therefore, segments can be actually composed of smaller subsegments that cannot be discovered due to the selected parameterization.

Furthermore, evaluating significant differences between segmentations has the limitations of the underlying logistic regression models; one of the main limitations is, for example, the assumption of linear response. In addition, the segments are clustered with an FNMM algorithm, which assumes the data as a combination of multivariate Gaussians. This seems a reasonable assumption in the case of chromosomal movements.

Similarly, with regard to motif discovery, the window-length—the size of motifs to be discovered—must be specified manually. This will affect the number, type, and relevance of the features that are discovered, making them extremely context-dependent. Furthermore, interpreting the results of motif discovery is trivial in the case of 1D time-series; higher dimensional cases may require additional effort.

#### 2.7.3. Causality Graphs

Regarding the discovery of causal relationships using graphical models, there is a fundamental premise: these analyses should not replace additional experimental work to validate causal relationships between variables. Furthermore, classical methods for causality analysis, such as the PC algorithm, were not designed from the ground-up to handle time-series as input data. Extra care must be taken when selecting input variables. In this regard, Glymour et al. review some of the common issues when using causality discovery approaches for time-series analysis [63].

To use the causality-discovery module included in *ChroMo*, the user must provide three input parameters: (i) the *p*-value threshold to consider a significant relation (<0.01 by default); (ii) the number of lags; in general, increasing the number of explored lags may increase the number of discovered causal relationships; this is mainly the case with VLTE, while not necessarily revealing meaningful relationships, depending on the biological question. Therefore, the time scales of the study and whether large or small time lags are significant should be considered. For example, it may be worth exploring how chromosome morphology behaves with respect to movement 10 time units (e.g., minutes) later, which may reveal some causal relationships. Exploring lags up to 50 min may reveal more causal relationships, but in the context of our question, it is likely noise. (iii) Another parameter that must be specified is the presence of connection, which is only a filter for the percentage of cases in which a causal relationship was detected, above a threshold of significance (i.e., a *p*-value < 0.01). In general, a threshold >0.8 will show strongly conserved causal relationships discovered in the data; lower thresholds may still be reasonable, depending on how the existence of a causal relationship is conserved across replicates. If a relationship is only found in 50% of the cases, which correspond exactly to all cells of the same biological replicate, the result has a very high uncertainty. For this purpose, in addition to displaying the causality graph, *ChroMo* provides the underlying adjacency matrix with all pairwise connection presences between variables.

### 2.8. Strains, Growth Conditions, and Meiosis Induction

Strains used throughout this study are specified in Appendix A. Strains were obtained by standard genetics techniques [64]. Homothallic (*h90*) haploid strains were grown on YES medium plates at 32 °C. Then, biomass was plated on an SPA agar plate incubating to 6 h at 28 °C. Then 50 µL of 0.2 mg/mL soybean lectin (Sigma Aldrich, St. Louis, MO, USA) were put in the centre of a 35 mm glass culture micro-dish microscopy (Ibidi Gmbh, Gräfelfing, Germany) and let sit for 2 min. Biomass was taken with a sterile toothpick and re-suspended in 200 µL MilliQ, and, after lectin was recovered from the plate and let dry, 100 µL of the cell suspension was put in the centre of the plate for 4 min. Eventually, EMM minimal medium was used to perform successive washes of the remaining biomass and to fill the plate with up to 3 mL.

### 2.9. Fluorescence Microscopy, Image Processing, and Analysis

Time-lapse image data was obtained using a DeltaVision widefield microscope system (Applied Precision, Issaquah, WA, USA), equipped with a Photometrics CCD CoolSnap HQ camera, a UV filter, and an Environmental Chamber set at a constant temperature of 28 °C, checked for focus stability 30 min before filming. Images were taken with a 100×, 1.4 NA oil immersion objective, every minute for 3 h over 20 z-planes at a 0.4 µm step size. Exposure time and transmittance values were 100 ms/32% respectively, for fluorescence channels, and 50 ms/50% for the DIC channel. Light at 436/10 nm wavelength was used to excite CFP-tagged histone Hht1.

Live-fluorescence microscopy files were processed using ImageJ. First, maximum slice intensity z-projections were obtained, and then background removal and drifting correction were applied using StackReg [65]. After Otsu’s thresholding and selection of the regions of interest (ROIs) containing meiotic cells, signals from chromosomes were treated as binary large objects (blobs); coordinates and morphology of blobs were obtained, using the tool *Measure*, and latter connected, frame-to-frame, into particle tracks using the *trackpy* package with default parameters [66]. Tracks were normalised with respect to time-point 0, this being the moment when two clear chromosome blobs are distinguishable. In *ChroMo*, the option *Last is common and zero* was selected.

## 3. Results

### 3.1. First Steps with ChroMo

We have developed the open-source web application *ChroMo* as a precise and unsupervised method for exploring meiotic CMs from live-imaging data to find patterns, microvariations, and causal relations. *ChroMo* is accessible in two ways: as a freely available, browser-based version at https://chromo.cloud (accessed on 4 August 2021); and as a standalone application that can be run locally.

The first step after launching *ChroMo* is to select a data source containing the time-series variables or descriptors obtained from fluorescence live-imaging of meiotic cells (e.g., position and shape of the chromosomes). This information can be obtained from imaging files either manually or via software like ImageJ. The data source can be uploaded from a local file, or loaded as a remote resource (e.g., from cloud storage) in any of the supported formats (e.g., CSV, TSV, XLSX) (Supplementary Figure S1). *ChroMo* was initially designed to explore the CMs during meiotic prophase in fission yeast, but it also supports other types of movement (e.g., it can follow the behaviour of the spindle pole body, motor of nuclear motion in *S. pombe*) and organisms, as well as other meiotic stages, for instance, the two rounds of chromosome segregations in MI and MII. As such, the descriptors must be chosen according to the type of CM; for instance, meiotic prophase in *S. pombe* is mostly characterized by the change in nuclear morphology during CM. For this reason, we established seven descriptors in two groups: (i) the chromosome morphology: circularity, convexity, minor axis, major axis and area; and (ii) the change in position with respect to the *x* and *y* axes: linear velocity of the gravity centre of the chromosome mass, and angular velocity (the change in angular orientation across time).

Users can select descriptors of interest (for example, linear velocity) to act as the main variables for their analysis. *ChroMo* provides several well-known ways of standardizing variables, such as 0-to-1 or Z-score normalization. Furthermore, if not all cells are exactly in the same position (i.e., different rotations), the main coordinates can be automatically transformed to ensure that all cells have comparable axes. Finally, time-point normalization must be indicated, that is, whether the first or the last point of the track is the common zero. This is important for later testing of significant differences in segment composition, motif analysis, and correct plotting of data.

### 3.2. ChroMo Provides More Detailed Information about Fission Yeast Meiotic Prophase

One of the aims of *ChroMo* is to find patterns in meiotic CMs that are difficult to observe directly. *ChroMo* can identify and cluster patterns of movement in an unsupervised manner and test the significance of their presence. We validated this using CM in fission yeast, which can be easily followed via live imaging by endogenously tagging one of the two copies of histone 3 with cyan fluorescent protein (CFP). Since nuclear movements are transmitted to the chromosomes by the telomere bouquet, we could visualize the dynamics of CM during prophase in *wt* settings using time-course analysis with one-minute intervals (Figure 1a). We used the seven *ChroMo* descriptors to describe the chromosomes throughout meiotic prophase in terms of the trajectory of their centre of mass and their morphology (Figure 1b). The last measurement of each cell before two masses of chromosomes were visible (referred to hereafter as MI) was selected as the common zero, and we uploaded this data to *ChroMo* as a CSV file. *ChroMo* then applies a two-step method: (i) segments are discovered in each of the individual time-series for any of the variables specified (Figure 1c), and (ii) these segments are collected and classified into behavioural clusters (Figure 1d). In short, clusters are sub-sequences of the whole CM that have a similar behaviour across all cells according to a user-selected set of descriptors. *ChroMo* can also test whether this segmentation is significantly different between groups, e.g., between specific mutants and *wt* settings.

The *ChroMo* analysis identified four clusters across meiotic prophase in our *wt* dataset; this is two more than the current model, which describes two phases of meiotic prophase in fission yeast (horsetail and post-horsetail) [31]. Upon further inspection of the spectrograms produced by *ChroMo*, we can describe the average behaviour of each of the four clusters (Figure 1c,d). Cluster 1 is characterized by the well-known oscillatory movement with a period around 6 min. Cluster 2 has similar characteristics but higher periodicity, which would still be considered horsetail movement according to the current two-stages model. However, cluster 2 is a consistently slower movement than cluster 1, taking place 130 to 90 min before MI. Cluster 3 illustrates the transition from the oscillatory movement in cluster 1 and 2 to the confined-Brownian or post-horsetail movement in cluster 4. Consistently, *ChroMo* also identified four clusters across meiotic prophase considering chromosome morphology descriptors (Supplementary Figure S2a).

To support this analysis, *ChroMo* generates diverse types of plots, such as representing each descriptor with respect to its distribution and its average time progression (Supplementary Figure S2b). Analysis of segmentation and clustering is one of the powerful tools available in *ChroMo*, allowing users to discover not only differences in the time-ensemble of meiotic prophase, but also smaller, automatically detected segments and clusters that may represent different and conserved behaviours.

### 3.3. ChroMo Detects Causal Relations in CM Time-Series

As we found in our *wt* dataset, chromosome movement and morphology in *S. pombe* oscillate dramatically during meiotic prophase. The amount of movement and the variability in morphology are crucial for the correct pairing of homologous chromosomes [67]. Hence, we studied chromosome position and morphology to assess the possibility of two-way interactions between these descriptors. This would allow us to hypothesize about unexplored relationships between chromosome organization and their movement during prophase.

*ChroMo* includes a set of algorithms that allow causality relationships to be studied across all user-defined variables, that is, whether changes in one variable at a time *t* can explain changes in other variable at time *t* + τ, with τ ≥ 0, where τ is the time lag. Three causality discovery approaches are included: (i) the Peter-Clark (PC) algorithm, (ii) variable-lag transfer entropy (VLTE), and (iii) variable-lag Granger causality (VLGC) (see Methods). These algorithms aim to assess how the amount of information in one variable, A, explains changes in another variable, B, better than B explains itself. This approach is stricter than cross-correlation analysis, which can determine if two variables behave similarly but does not necessarily support causation. Moreover, PC requires stationarity of the variables of interest; in brief, a variable is considered stationary when its properties depend not on when it is observed, but rather on an underlying fixed distribution. VLTE and VLGC do not strongly assume stationarity, so they are ideal for assaying biological processes that may change their driving features across time, like CM in meiotic prophase.

**Figure 2:**
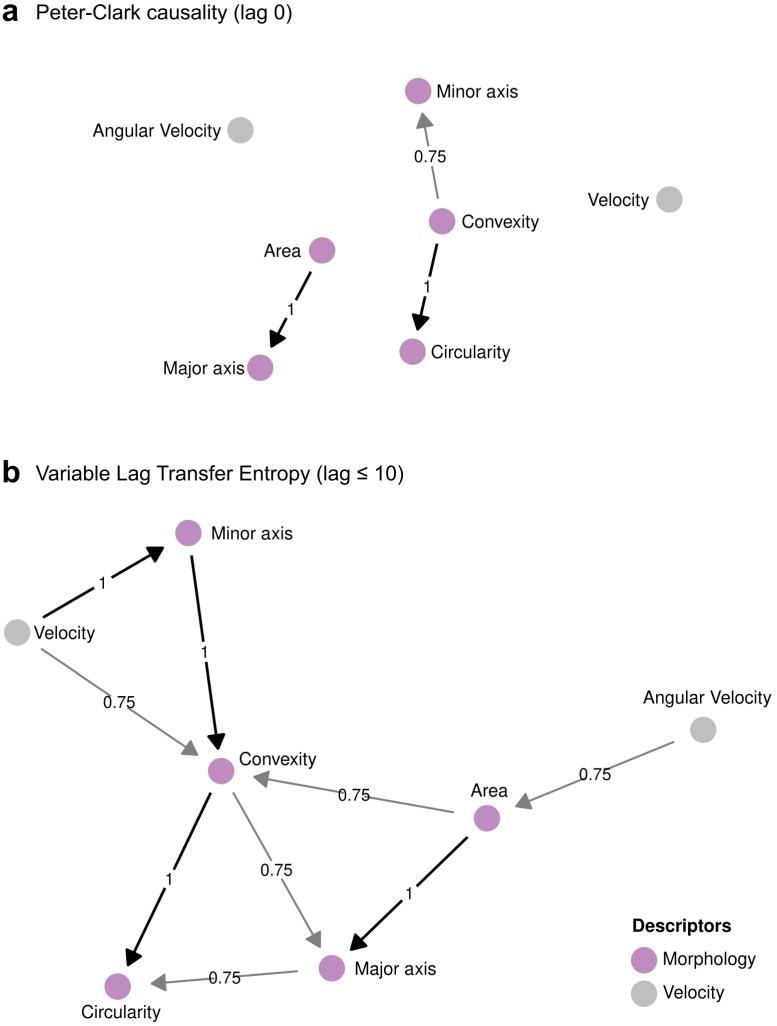
Causality analysis between morphology and movement variables. Directed Graphs (DG) for the (**a**) PC and (**b**) VLTE algorithms are depicted with a conservation of 75%; for the VLTE algorithm, the maximum selected lag was 10; in both cases, a *p*-value < 0.05 was considered significant. Nodes are all variables studied in the causality analysis. In grey, velocity descriptors; in pink, morphology descriptors. Edges indicate if nodes are connected by a significant causality relation, and causal direction (arrowhead). Edge weights represent the conservation of the relation across all individual cases, i.e., how many times a significant relation appears on the dataset. Weights are shown on edges and represented by line width and grayscale intensity.

We used *ChroMo* to perform causality analysis with these algorithms across our meiotic CM experimental *wt* dataset and aimed to detect relationships between morphology and position descriptors. In this way, a causal relationship is considered feasible if the results of the algorithm are statistically significant for more than a certain percentage (in this case, a presence threshold of 75%) of cells (Figure 2). For example, let A and B be two time-series; a relationship A → B with a weight 1 means that A significantly causes B in 100% of cases. Both PC and VLTE identified strong relationships between morphology descriptors with a time lag up of to 10 min (Figure 2a,b); however, the VLTE framework seems more appropriate to this specific case, as this dataset is not strictly stationary. Additionally, VLTE was more informative than PC for this dataset (Figure 2b). VLTE discovered the relationship *Velocity* *→* *(Minor axis, Convexity)* in the network, meaning that changes in velocity affect morphology up to 10 min later. This fits with the expectation that faster CM is linked to a horsetail shape, whereas slower CM leads to a more rounded shape, i.e., when chromosomes are in the equator or during the post-horsetail stage. When the presence threshold is reduced to 50%, both PC and VLTE discover morphology → velocity relationships (Supplementary Figure S3). This suggests that changes in how chromosomes are organized may affect the velocities at which they are moved, albeit in a smaller percentage of cells.

### 3.4. Tuning up ChroMo Analysis with a Synthetic Dataset

One important limitation in studies of chromosome dynamics during meiotic prophase is the difficulty in filming meiotic cells in vivo. Other limitations include the need for large sample sizes and the fact that mutants must be analysed one by one after live fluorescence microscopy. Additionally, although it is common to use time-ensemble descriptors that are suitable for detecting global differences; e.g., CM being slower on average in a mutant than in *wt*, it can be more difficult to investigate events of shorter duration. *ChroMo* was designed to overcome these limitations. It takes advantage of current developments in data mining to automatically annotate behaviours and to discover potentially unknown phenotypes. In this way, *ChroMo* increases both analysis power and reproducibility.

**Figure 3:**
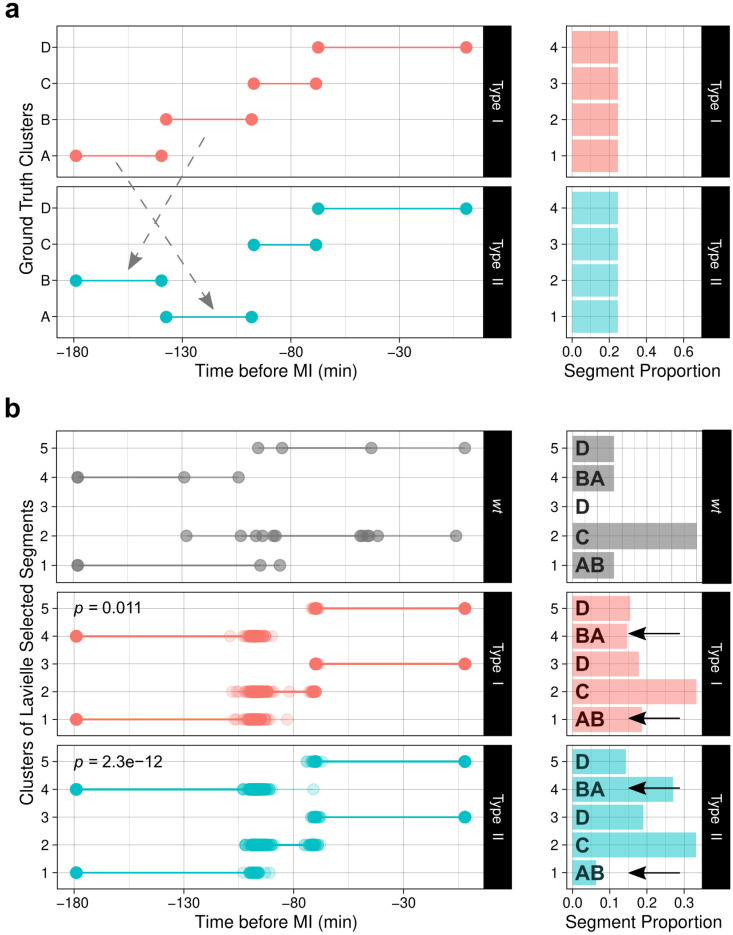
Segmentation of *wt* and synthetic chromosome movement datasets. The 6 *wt* tracks and 200 simulations of 180 samples each were used throughout this analysis: (**a**) On the left, segment-plot of the ground-truth segmentation for the simulated time-series. On the right, presence proportion of each segment in the dataset. (**b**) On the left, segment-plot of a proposed segmentation after *segclust2d*’s algorithm with Lavielle’s criterion selection: from top to bottom, experimental *wt* CM data, Type I and II synthetic tracks. Reported *p*-values (top left of each segment-plot) for the ANOVA after Logistic Regression (LR). LR models in the comparison are the interaction between cluster type and duration, and the intercept-only model, between the baseline case (*wt*) and the two synthetic datasets. On the right, relative count of cluster presence across the datasets. Time scales are adjusted w.r.t. the beginning of MI.

To identify the presence of subtle variations of CM that are not detectable using time-ensemble descriptors, we propose two piecewise functions (synthetic Type I and II) inspired by the oscillatory nature of the segments and clusters identified in our *wt* dataset (Supplementary Figure S4). Type I is designed to yield a synthetic dataset with a trajectory close to that observed in the *wt* dataset, in four equivalent movement stages (clusters A, B, C, and D). Type II is configured to invert the first and second movement stages, so cluster B comes before A (Figure 3a). Common time-ensemble descriptors of the time-series, such as velocity profiles, show no significant difference between groups even with a sample size of 400 tracks (Supplementary Figure S5), which shows how a segment-oriented approach might be beneficial. In this regard, *ChroMo* includes two well-known segment-discovery libraries: *segclust2d* for one- or two-dimensional problems, and *segmenTier* for higher-dimensional series. By applying the *segclust2d* algorithm to our synthetic dataset, *ChroMo* identified five clusters: 1 and 4 correspond to clusters A and B, respectively; cluster 2 corresponds to cluster C; and clusters 3 and 5 correspond to cluster D (Figure 3b).

The order of clusters A and B is switched between Type I and Type II sequences, which is reflected in the relative abundance of clusters 1 and 4. Furthermore, we fitted several logistic regression models (see Methods) to test whether segment location and cluster composition is different across Type I and Type II sequences and obtained significant evidence that it is (Figure 3b). To determine the sample size needed, we assayed the statistical power (1−β) of the logistic regression models. In brief, statistical power is the probability of rejecting the null hypothesis of a test when the alternative is true, i.e., the proportion of times that the difference between segmentations is considered significant, given that the ground truth is in fact a different segmentation. A sample size of at least 60 per category was needed to reach (1−β)=0.8 for this specific ground truth, while power for global descriptors in same settings did not reach (1−β)=0.5. Using this approach, *ChroMo* could recapitulate the true differences in the sequential nature of synthetic trajectories across groups. This is potentially useful for future studies that aim to find features of meiotic CM in mutants rather than in time-ensemble analysis.

### 3.5. ChroMo Performs Segmented and Time-Wise Analysis of Spectrum and Velocities

*ChroMo*’s automatic segmentation and clustering approach finds different behavioural modes with higher resolution in time-series compared to time-ensemble analysis. However, it is not clear why ground-truth clusters A and B were considered inside whole segments or how they look on average, as traditional density plots of the proposed segmentation do not show differences between clusters 1 and 4 (Figure 3). Thus, timewise visualizations could potentially be useful to find out what the detected differences look like in reality, in density and across time.

**Figure 4:**
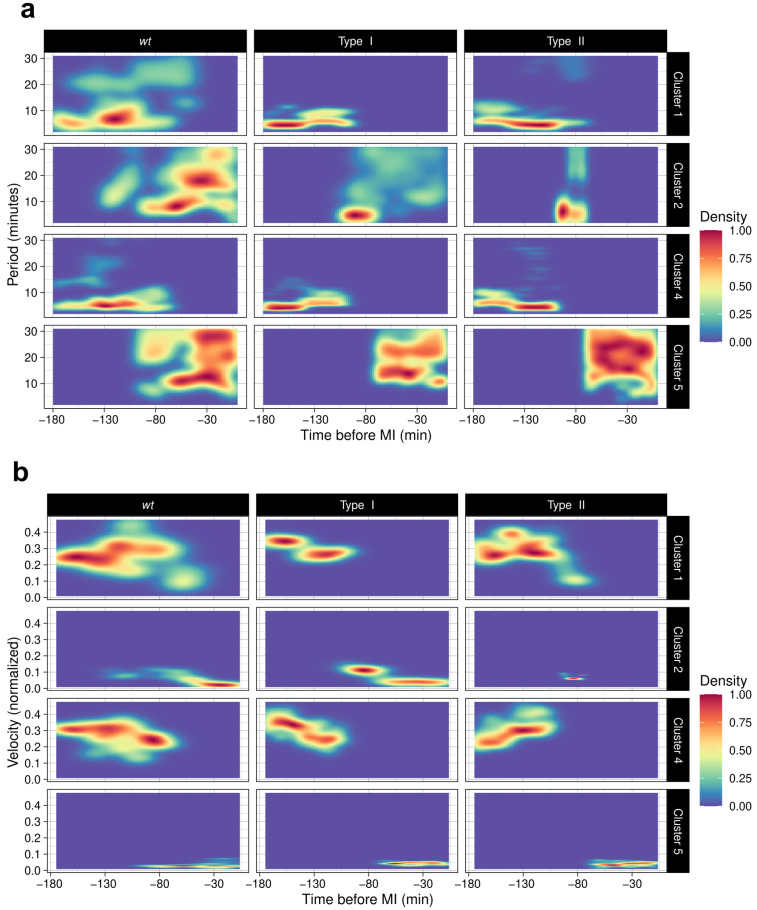
Temporal assessment of spectrum and velocity, per cluster, across *wt*, Type I and II (synthetic) datasets. Heatmaps, faceted by experimental group and behavioural cluster, for (**a**) data-ensemble spectral densities; XY axes represent Time and Period in minutes, respectively. (**b**) Heatmaps of velocities across the dataset; XY axes represent Time and Velocity, in minutes and normalized as AU/minute. Colour level represents the normalized density for period or velocity.

We used *ChroMo* to generate heatmap plots for spectral and velocity features to visualize behaviour across groups and clusters, on average and with respect to time. In Figure 3, clusters 1 and 4 are each composed of two smaller parts (namely, A and B) in opposite order, as expected given the ground truth; also, Type I is mostly similar to the *wt*. In terms of spectral density, cluster 4 is different across Type I and Type II sequences, with two distinct frequency domain behaviours at −180 to −130 and −130 to −80 min before MI, respectively (Figure 4a). The same applies to velocity; in the case of cluster 4 in Type I, velocity is higher and then lower at the same times as shown in the spectrograms, with the opposite situation in Type II sequences. This means that cluster 1, which is mostly present in Type I, has higher velocity and narrower spectral density (the case for cluster B) first, then lower velocity and wider spectral density (the case for cluster A), for a final order of *BA* (Figure 4b). In Type II, the opposite is true for a cluster with order *AB*, as expected given the ground truths for this synthetic dataset (Figure 4). In summary, these *ChroMo* visualizations illustrate the similarity between synthetic and experimental datasets, and better show different timewise patterns compared to the classical time-ensemble density plots.

### 3.6. ChroMo Uses Motifs to Add a Complexity and Detail Layer to Behavioural Segments

Segments generally describe behavioural trends in distribution for specific time windows. However, this concept of time-series analysis does not provide information about the conservation of specific value sequences. For example, we may want to assess if specific movement patterns (values), rather than similar velocity profiles (distribution), are conserved in specific conditions, as different movement sequences may yield equivalent velocity profiles. Motif (discord) analysis can help to solve this challenge by considering value sequences and finding the most common patterns (and discrepancies) that appear at any location. Consequently, it is also a comprehensive way of visualizing the average behaviour of time-series, in terms of their actual values, rather than providing summary distribution visualizations.

**Figure 5:**
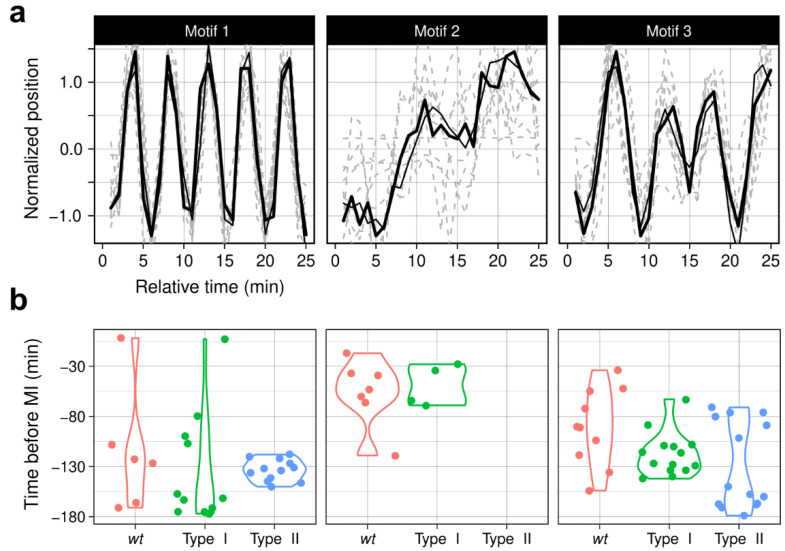
Discovery of motifs in the dataset, for *wt*, Type I and II (synthetic) datasets. (**a**) 3 motifs, discovered using a time-window of 25 min, are shown. In each motif plot, a maximum of 100-top matches are aligned. (**b**) violin-plot illustrating the time distribution of each motif for each group.

*ChroMo* covers this kind of analysis by including algorithms for Matrix Profile generation and motif discovery. Using our synthetic trajectories dataset, we can explore the average appearance of all sequences at once, summarized by the top three motifs and their locations (Figure 5). Analysis of these motifs in our *wt* dataset shows that there are two types of oscillatory movement with significantly different time locations; motif 1 has a higher average frequency than motif 3, which has a less sine-like oscillation. This is consistent with the greater periodicity and velocity bands seen between 130 to 80 min before MI in the previous section (Figure 4).

By definition, no differences in motif location are expected between synthetic Type I and *wt* data. On the other hand, Type II was specifically created to be different from Type I. As expected, motifs 1 and 3 have significantly different distributions across the synthetic datasets. Motif 1, as in the *wt* setting, is present at the beginning of Type I sequences (−180 to −130) but later in Type II sequences (−130 to −80). However, the opposite is true for motif 3. This further supports and illustrates the ground-truth segmentation, as Motif 1 better fits with cluster A, and Motif 3 with cluster B (Figure 5b).

Motif analysis is a tool to visualize the most common specific sequences of values rather than summary distributions. In our case, a window length of 25 min was sufficient to explore the differences in our dataset, but different window lengths can be tuned to exploit diverse patterns in other biological datasets; longer time windows may reveal behaviours conserved at extended time scales, e.g., stages of the cell cycle, while shorter windows may show patterns that occur at smaller time scales, e.g., as responses to events other than the mechanisms driving CM.

### 3.7. ChroMo Finds Undisclosed Features on Known Strains

Once we explored the potential of *ChroMo* with experimental and synthetic data, we sought to gain insight into the biological meaning of the results. We analysed CM trajectory and morphology tracks from two *S. pombe* deletion mutants for (i) the meiotic-specific microtubule-organizing centre *Hrs1*, also known as *Mcp6* [48,68], and (ii) the ATP-dependent DNA helicase *Rdh54* [69]. Loss of *hrs1* abolishes the strong horsetail movement [46,48,70], whereas on the other hand, loss of *rdh54* leads to defects in homologous recombination and prolongs the duration of the post-horsetail stage [31,69]. These proteins, which have roles in two distinct aspects of the meiotic prophase, are good candidates to exemplify the search for uncharacterized movement patterns or causality networks.

#### 3.7.1. ChroMo Shows That the Oscillatory Movement Patterns Are Conserved in the *hrs1Δ* Strain

**Figure 6:**
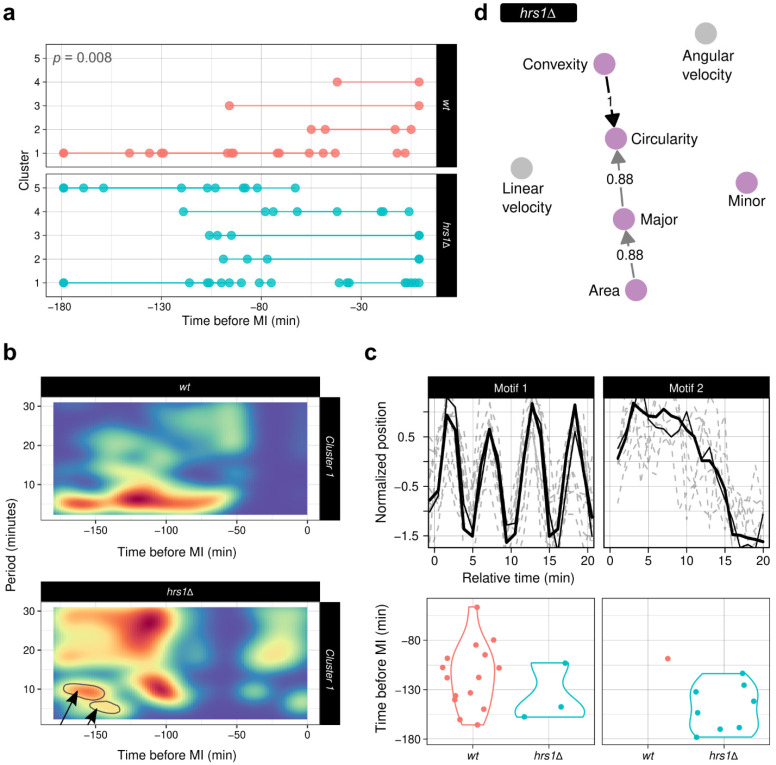
*ChroMo* analysis of CM in the *hrs1Δ* strain highlights an oscillatory movement. (**a**) Segment-plots of the unsupervised segmentation after *segclust2d*’s algorithm with Lavielle’s criterion selection, for linear and angular velocities calculated from rotated 2D trajectories. Top left, reported *p*-value for the ANOVA after LR between *wt* and *hrs1Δ*, comparing the interaction between cluster type and duration, and the intercept-only model. (**b**) Heatmap-spectrograms for cluster 1, across *wt* and the mutant. Black arrows and outlines inside the plot indicate the conservation of oscillatory behaviour around the 10 min period, early in meiotic prophase. (**c**) Top-2 discovered motifs, using a time-window of 20 min, are shown. In each motif plot, a maximum of 100-top matches is aligned. Below, a violin-plot illustrates the time distribution of each motif for all groups being analysed. (**d**) DG for the causality relations found by VLTE algorithm with a maximum lag of 10 and a presence threshold of 80%. Pink nodes illustrate morphology variables; grey edges illustrate movement variables.

We compared trajectory and morphology tracks that were reconstructed from the Hht1-CFP fluorescent signal in *hrs1Δ* and *wt* cells. Segmentation of both angular and linear velocities showed five clusters for *hrs1Δ* that were significantly different to those from *wt* in both composition and duration (Figure 6a). For cluster 1, spectral analysis showed a mostly noise-like spectrum, as CMs are mostly abolished in this mutant. However, a band around the 10-min period showed that oscillatory movement—with lower frequency than in *wt* settings—may be conserved between around 150 and 100 min before MI (Figure 6b). Motif analysis supported the conservation of some oscillatory movement through motif 1, which was detected at this same time window (Figure 6c). These results suggest that even though velocity is much lower in the absence of Hrs1, oscillatory movement is not completely lost. Regarding VLTE causality networks, *Trajectory* → *Morphology* relationships are lost in *hrs1Δ* compared with *wt* (Figure 6d), even with a presence threshold of 0.6; however, the relationship *Major* → *Angular velocity* is present in 62% of cases. These results illustrate how abolishing fast CM may not necessarily involve a complete loss of its oscillatory behaviour. They also support the notion that slow spindle pole body (SPB)-driven CM in fission yeast does not have a significant role in altering chromosome morphology.

#### 3.7.2. The *rdh54* Deletion Leads to Different Oscillatory Movements

**Figure 7:**
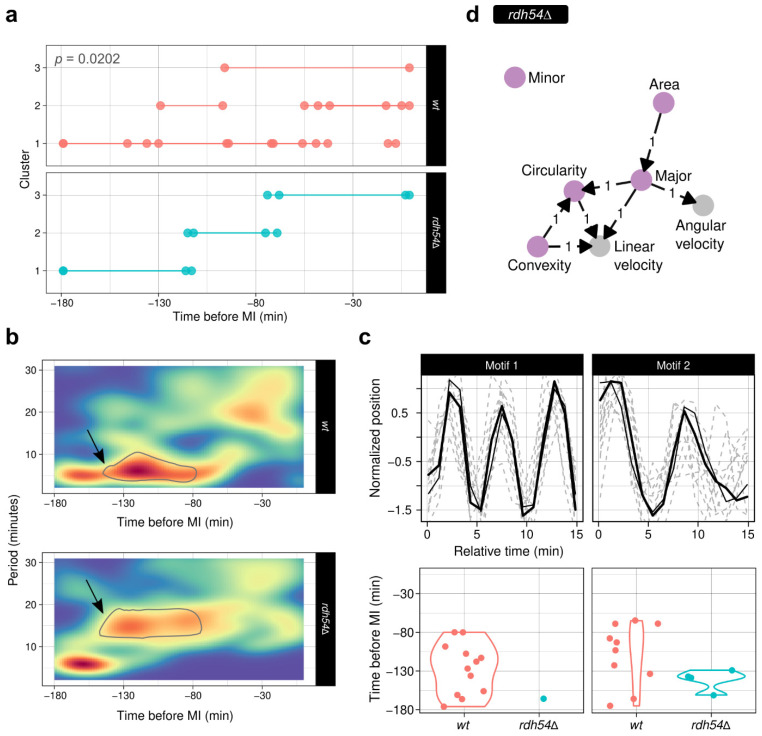
*ChroMo* analysis of CM in the *rdh54Δ* mutant reveals difference in the oscillatory movement. Analogously to Figure 6, (**a**) segment-plots for the unsupervised segmentation of velocity descriptors across both datasets. Top left, reported *p*-value for the ANOVA after LR between *wt* and *rdh54Δ.* (**b**) Global, non clusterised, heatmap-spectrograms of position data for the *wt* and the *rdh54Δ* mutant. Black arrows and outlines inside the plot show the time range at which the oscillatory period changes between both strains. (**c**) Top-2 discovered motifs within a time-window of 15 min, and the corresponding distributions of their duration, below. (**d**) DG for the causality relations found by the VLTE algorithm with a maximum lag of 10 and a presence threshold of 80%.

For the *rdh54* mutant strain, *ChroMo* segmentation analysis found three clusters that were significantly different to those found for the *wt* case (Figure 7a). Spectrograms show how the frequency band between 110 and 80 min before MI is even broader than in the *wt* (Figure 7b), suggesting that the oscillatory movement in the mutant may be more unstable at that moment. To validate these findings, we performed motif analysis; for motif 1, this characteristic *wt* oscillation pattern is kept for the *rdh54Δ* case only during the temporal domain of cluster 1 (around 180 to 130 min before MI), whereas in *wt* settings it is present at around 180 to 80 min before MI; this is equivalent to the information provided by the spectrograms (Figure 7c). This suggests that the loss of Rdh54 might be responsible for these changes in the periodicity of the oscillations. Finally, VLTE analysis revealed strong (*Convexity, Circularity, Major*) → *Linear Velocity* and *Major* → *Angular velocity* relationships (Figure 7d). This, together with the differences in motif composition, implicates morphology in the broader oscillation spectra observed in trajectory; hence, possible changes in morphology, likely associated with defective homologues recombination, affect the velocity spectrum. Further work to define these possible connections is underway in our lab.

## 4. Discussion

The intense chromosome movements during meiotic prophase are crucial to promote pairing and recombination between homologous chromosomes. These movements are driven by cytoskeleton forces transmitted to the chromosomes by the telomere bouquet. The bouquet and movements are evolutionarily conserved across plants and opisthokonts and likely originated early in the origin of eukaryotes. Although mutations that compromise *wt*-like CM have been characterized in many model organisms, commonly used time-ensemble analysis might hide potentially interesting trajectory or morphology patterns that are hard to detect across meiotic prophase. Data mining and time-window approaches can overcome some of the limitations of time-ensemble analysis, but a platform combining unsupervised tools for this specific type of dataset was not available. We developed *ChroMo*—a comprehensive, reproducible, and high-throughput web application—to automatically perform this task using current time-ensemble approaches as well as new segment and motif-oriented strategies. *ChroMo* is a useful tool for discovering patterns of variable length, from larger sequences down to smaller, more infrequent subsequences. In this regard, *ChroMo* includes segment-discovery libraries, such as *segclust2d* and *segmenTier,* and motif detection through Matrix Profile calculation. Moreover, *ChroMo* is designed to better take advantage of the information obtained during time-lapse experiments. It includes easy-to-use interfaces for three well-established causality analysis algorithms (PC, VLTE, and VLGC), which allow users to explore two-way causal relationships between any input variables, such as chromosome morphology and movement.

We have validated our analyses with synthetic data, based on previous knowledge of CM in a *wt* background. These analyses were a fundamental first step to assay the robustness of *ChroMo*, determine its sample size requirements, and illustrate the potential of the analysis and visualizations. Using *ChroMo*, we gained information about patterns of movement in *wt* settings by identifying, among others features, four clusters during the meiotic prophase instead of the two canonical stages, horsetail and post-horsetail movements. We then studied two mutants that are known to affect the meiotic CM: (i) *hrs1*Δ, in which the strong nuclear movements are markedly reduced, and (ii) *rdh54*Δ, in which cells show a prolonged phase of post-horsetail movement. *ChroMo* revealed novel information about both mutants. Although we confirmed that the dramatic nuclear oscillations observed in the *wt* are abolished in *hrs1*Δ, this mutant had some oscillatory behaviour with a higher average periodicity than that in the *wt*; this suggests that movement periodicity but not linear velocity is conserved in *hrs1*Δ settings. For the *rdh54*Δ mutant the oscillatory spectrum during the time corresponding to cluster 1 was broader than in the *wt*, which points to a higher average instability of CM. This kind of movement, different to that found in the *wt*, might have biological meaning: deletion of *rdh54* increases DNA damage accumulation during meiotic prophase, and DNA damage may induce alterations in chromosome morphology [51]. Accordingly, cross-studying movement and morphology via VLTE causality analysis revealed a *Convexity → Velocity* relationship that was stronger in *rdh54*Δ than in *wt*. This suggests that changes in morphology, especially in convexity, might provoke changes in how trajectory progresses later on. We are developing future studies to confirm these results in our lab.

*ChroMo* is an open-source web application that is freely available at https://chromo.cloud (accessed on 4 August 2021). To help users explore the capabilities of *ChroMo* (Table 1), the application includes experimental examples of meiotic prophase in fission yeast and synthetic datasets. Users can familiarize themselves with *ChroMo* using these datasets. Further improvements in the application will extend its use for the characterization of meiotic chromosome dynamics, not only in yeast but in metazoans in general.

## Figures and Tables

**Table 1:** Description of *ChroMo* analysis modules, functions, and their general use cases.

Module	Function	Description	Use-Cases
Segments	Discovery	Performs time-series segmentation, per particle ID and per group, and clusters all discovered segments into a common pool, visualized as a clustered *segment plot*. Then, assesses if the cluster composition (location, abundance) is a good predictor of group using logistic regression models.	Discover different behavioural modes in 2D or 3D trajectories of tracked particles. For chromosome movement, a good criterion to analyse velocity and trajectory in the main axis of movement is the *Lavielle* method. Discover different behavioural modes related to nuclear morphology. A good approach is to use the *Multivariate* method with as many morphology variables as possible.
	Cluster Summary	Displays a table-like summary of the discovered clusters, and the corresponding features used for clustering.	---
	Spectrum	Calculation of spectral characteristics of the main coordinate variable (only in 1D), per particle and group ID. Displays global and per-cluster spectral densities, as well as heatmaps of spectral density across time, global and per discovered cluster—needs prior segmentation of data with the function *Discovery*—. Provides a summary with statistics to assess whether the spectrum is significantly different between strains.	Analyse the periodicity of the trajectory tracks of chromosome movement, in general and per behavioural mode. Study if changes in morphology or amount of signal follow some periodicity.
	Distribution	Calculates the empirical distribution (probability density) of any selected numerical variable (column of the dataset) and displays it on violin and density plots, for the whole time-series, per group, or per group and discovered behavioural mode. Additionally, it calculates the 2D density of the selected feature over time. It is possible to set up a moving average to smooth the data. Provides a summary with statistics to assess whether the probability density is significantly different between strains.	Study how different morphology descriptors vary depending on the clustered behavioural mode. Explore how velocities are distributed over time, on average for all particles, per group and cluster.
	Displacement	Calculate the Mean Squared Displacement (MSD) of the data selected as *coordinate variables,* per group, and per discovered cluster.	Visually assess, globally and per behavioural segment, how chromosome movement may correspond to different types of motion, e.g., directed, Brownian, super-diffusion.
	Individual	Tools to visualize the time-series of each particle, as well as the individual spectrograms for any variable.	---
Motifs	Global	After selecting a variable (1D) and groups to analyse, motifs (and discords) are calculated for the concatenated time-series. It is possible to parametrise the window-length for which motifs will be discovered, as well as other tuneable parameters (see *tsmp* reference guide on these [71].	Explore patterns for the same variable across subjects; for example, relative to the main axis of motion. If the time-series always have identical start and end parts, the connection points can be detected as motifs.
	Per cluster	Analogous to *Global* motif analysis, for each discovered behavioural cluster. Displays the distribution of discovered motifs across time.	Visually explore whether different behavioural segments have different patterns that may explain their differences.
Causality	PC-alg VLTE VLGC	Calculates a causality graph using the PC-algorithm, Variable-Lag Transfer Entropy, or Variable-Lag Granger Causality, for selected variables, per particle ID and per group. Then, it builds a global graph of all the observed relationships in all the particles. Configurable parameters are the significance threshold (*p*-value) to consider a relationship, the maximum lags to explore, and the presence of connection, with respect to how many times a relationship must appear in the dataset to be considered positive.	Explore the causal relationships that explain the mutual influence of chromosome morphology on movement.
	Correlation	Calculates a pairwise correlation between all selected variables and shows the corresponding scatterplots.	Study the dependencies between the morphology descriptors, for example, how the area is positive-linearly dependent on the major and minor axes.
	Matrix	Shows the adjacency matrix for the calculated causality graphs	---

## Data Availability

Synthetic and experimental datasets, source code, Docker images, and configuration files for Cloud deployment are available in https://github.com/danilexn/chromo (accessed on 4 August 2021).

## Supplementary Materials

The following are available online at www.mdpi.com/article/10.3390/cells10082013/s1, **Supplementary Figure S1: *ChroMo* user interface overview** (**a**) Input data sample format. The user is expected to load time-series data, with a column containing the time points (“frame” in our dataset). In addition to the specific variables of the study, the dataset must contain a column that indicates the ID of the particle (“particle” in our data) and the group to which it belongs (“label” in our data, represents the ID on our strain database). (**b**) Screenshot of the *ChroMo* data upload user interface, after choosing the included “Biological Example” dataset; this can be accessed via https://chromo.cloud, after starting any of the three instances—depending on CPU and memory resource allocation—, or after starting a local instance with the provided Docker image or source files, at https://github.com/danilexn/chromo (accessed on 4 August 2021). (**c**) Detail of the sidebar to adjust different settings in the input dataset. **Supplementary Figure S2: Segmentation protocols with positional- and morphology-derived descriptors.** (**a**) On the left, segment-plot of the segmentation for the velocities (above), and morphology descriptors (below). On the right, per segment-plot, presence proportion of each segment in the dataset. In both cases, segments are grouped by clusters (named from 1 to 4). (**b**) Showcase of different plots that can be automatically generated using *ChroMo*: 1D-density plots of all sequences for the spectrum (top left, LOWESS fitting shows the averages and deviations of the density per period) and MSD (bottom left). Violin- and box-plot (top right) of the calculated linear velocity for a *wt* strain, across all cells. Positions were normalized in the 0–1 range; hence velocities are non-dimensional with respect to the space, with unit *time^−1^.* Heatmaps of velocities (bottom right) across the *wt* dataset; XY axes represent Time and Velocity in minutes and normalized as *time^−1^*, respectively. **Supplementary Figure S3: Low-conservation causality analysis between morphology and movement variables.** Following the same structure as Main Figure 2, DG for the (**a**) PC and (**b**) VLTE algorithms are depicted with a conservation of 50%; for the VLTE algorithm, the maximum selected lag was 10; in both cases, a *p*-value < 0.05 was considered significant. **Supplementary Figure S4: Illustrating the design of synthetic data from the *wt* case.** (**a**) From individual tracks of CM in *wt* experiments, segments are studied (rectangles on top) and grouped into clusters (different grey levels). The properties of each segment, such as average velocity or amplitude and period, are studied and transformed into a phenomenological model, based on periodic functions of time with a random component (see Appendix A.2). Therefore, calculating the values of the functions at specific times, analogous to the discrete sampling of images, returns an array of values that correspond to synthetic data. (**b**) Heatmaps, grouped by behavioural cluster discovered after applying *ChroMo* segmentation, illustrates the similarities between actual CM data, and the simple phenomenological model, built to generate as many sequences as desired. **Supplementary Figure S5: Global versus per-cluster analysis of descriptors.** For our synthetic dataset, consisting of 400 series of 180 time samples each, no significant differences, except for the case of the spectrum, could be found between different types of ground-truth segmentation, namely Type I and Type II sequences. In every panel, analyses are performed globally (left) and per-segment (right), for (**a**) Normalized spectral 1D-density, (**b**) velocity profiles, in units time^−1^, and (**c**) Mean Squared Displacement.

## Appendix A

### Appendix A.1. Strains Used throughout the Study

**Table A1:** *Schizosaccharomyces pombe* strains used in this paper.

Strain	Feature	Genotype	Origin	References
AFA226	*wt*	h90 ade6-M210 his3-D1 leu1-32 Pnda3-mCherry-Atb2:aur1 Sid4-GFP:KanMX6 Hht1-CFP:his3	Lab stock	–
AFA824	*hrs1Δ*	h90 ade6-M210 his3-D1 leu1.32 Hht1-CFP:his3 Pnda3-mCherry-Atb2:aur1	JCF10361	[70]
AFA826	*rdh54Δ*	h90 ura4-D18 taz1-YFP:KanMX6 Hht1-CFP:ura4 Sid4-mCherry:NatMX6 mei4-mCherry:NatMX6 rdh54::zeoCV	KT2475	[31]

### Appendix A.2. Definition of Synthetic Time-Series

Segment composition for Type I and II synthetic time-series are expressed, in terms of particle position, *y_t_*. For Type I sequences:

(A1) $$y_{t}=\{\begin{array}{cc} u\rho_{1}sin\left(\rho_{2}t\right)+sin\left(\rho_{3}t\right)\left(-\frac{u}{4}+1\right)+W_{1} & 0\leq t\leq 40\\ \rho_{4}sin(\rho_{5}t)+\frac{3}{4}sin\left(\rho_{6}t\right)+W_{1} & 41\leq t\leq 80\\ \frac{\alpha sin\left(\rho_{7}t\right)}{t-t_{0}+\alpha }+W_{2} & 81\leq t\leq 135\\ y_{t-1}+W_{2} & 136\leq t\leq 180 \end{array}$$

For Type II sequences, an inversion between the first and second segments is characteristic:

(A2) $$y_{t}=\{\begin{array}{cc} \rho_{4}sin(\rho_{5}t)+\frac{3}{4}sin\left(\rho_{6}t\right)+W_{1} & 0\leq t\leq 40\\ u\rho_{1}sin\left(\rho_{2}t\right)+sin\left(\rho_{3}t\right)\left(-\frac{u}{4}+1\right)+W_{1} & 41\leq t\leq 80\\ \frac{\alpha sin\left(\rho_{7}t\right)}{t-t_{0}+\alpha }+W_{2} & 81\leq t\leq 135\\ y_{t-1}+W_{2} & 136\leq t\leq 180 \end{array}$$

The second dimension of trajectories, *x_t_*, has the following form in both cases:

(A3) $$x_{t}=\{\begin{array}{cc} x_{t-1}+W_{3} & 0\leq t\leq 135\\ x_{t-1}+W_{4} & 136\leq t\leq 180 \end{array}$$

with *ϵ* > 0 and the following manually chosen *α* = 2 and random variables:

$$\rho_{1}∼N\left(0.15,0.1\right),\rho_{2}∼N\left(0.75,0.05\right),\rho_{3}∼N\left(1.33,0.05\right)\;\;\;$$

$$\rho_{4}∼N\left(0.45,0.1\right),\rho_{5}∼N\left(0.65,0.05\right),\rho_{6}∼N\left(1,0.05\right)\;\;\;$$

$$\rho_{7}∼N\left(1.22,0.1\right),u∼Bin\left(1,0.3\right)\;\;\;$$

$$W_{1}∼N\left(0,0.2\right),W_{2}∼N\left(0,0.1\right),W_{3}∼N\left(0,0.05\right),W_{4}∼N\left(0,0.02\right)\;\;\;$$
