# Supplementary figures and images for: ChroMo, an Application for Unsupervised Analysis of Chromosome Movements in Meiosis
Source: Cells. 2021 Aug 6;10(8):2013. doi: 10.3390/cells10082013 (PMC8392469; doi:10.3390/cells10082013)

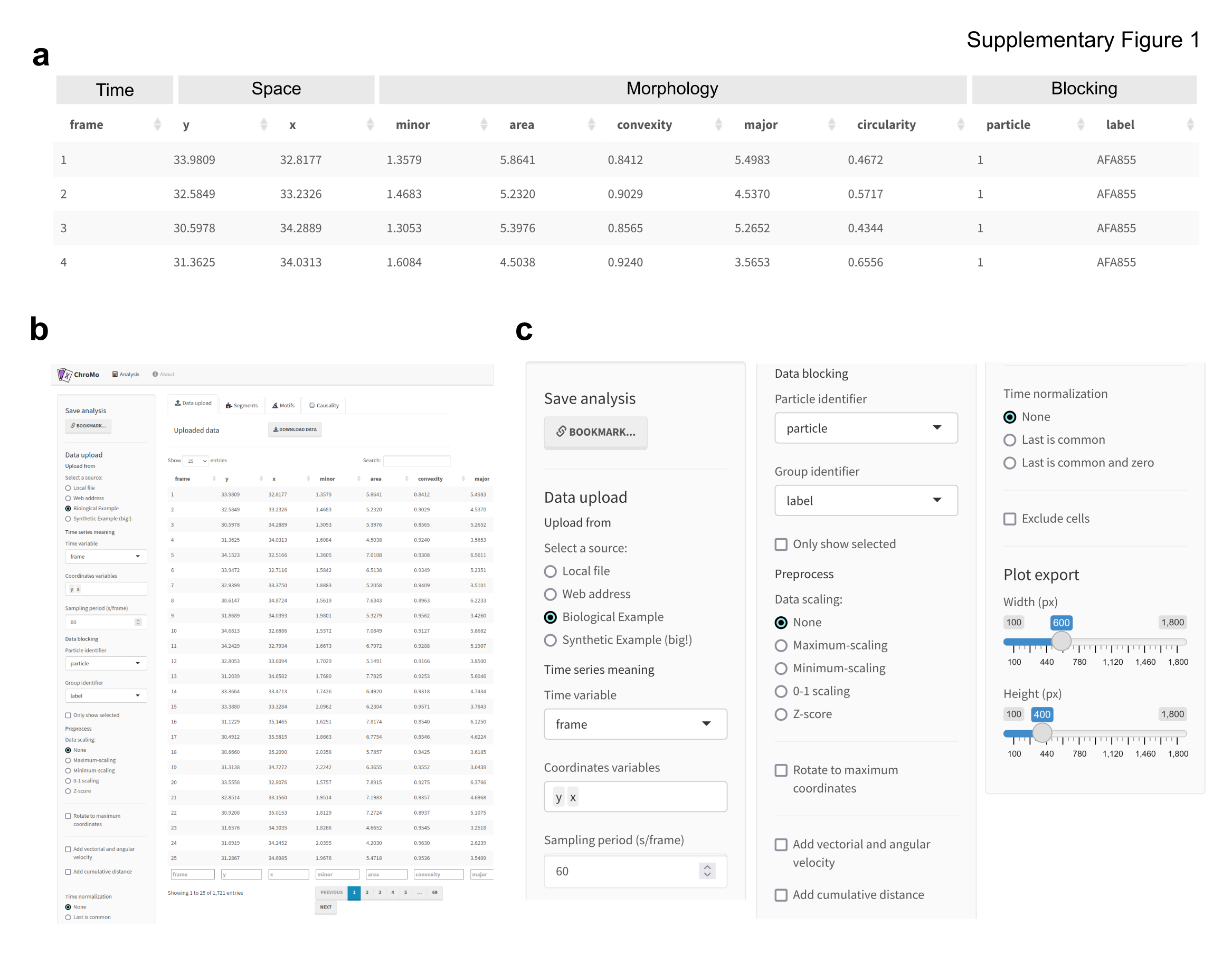

Supplement: Supplementary file 1 [file cells-10-02013-s001.zip › SuppFigure1.tiff]

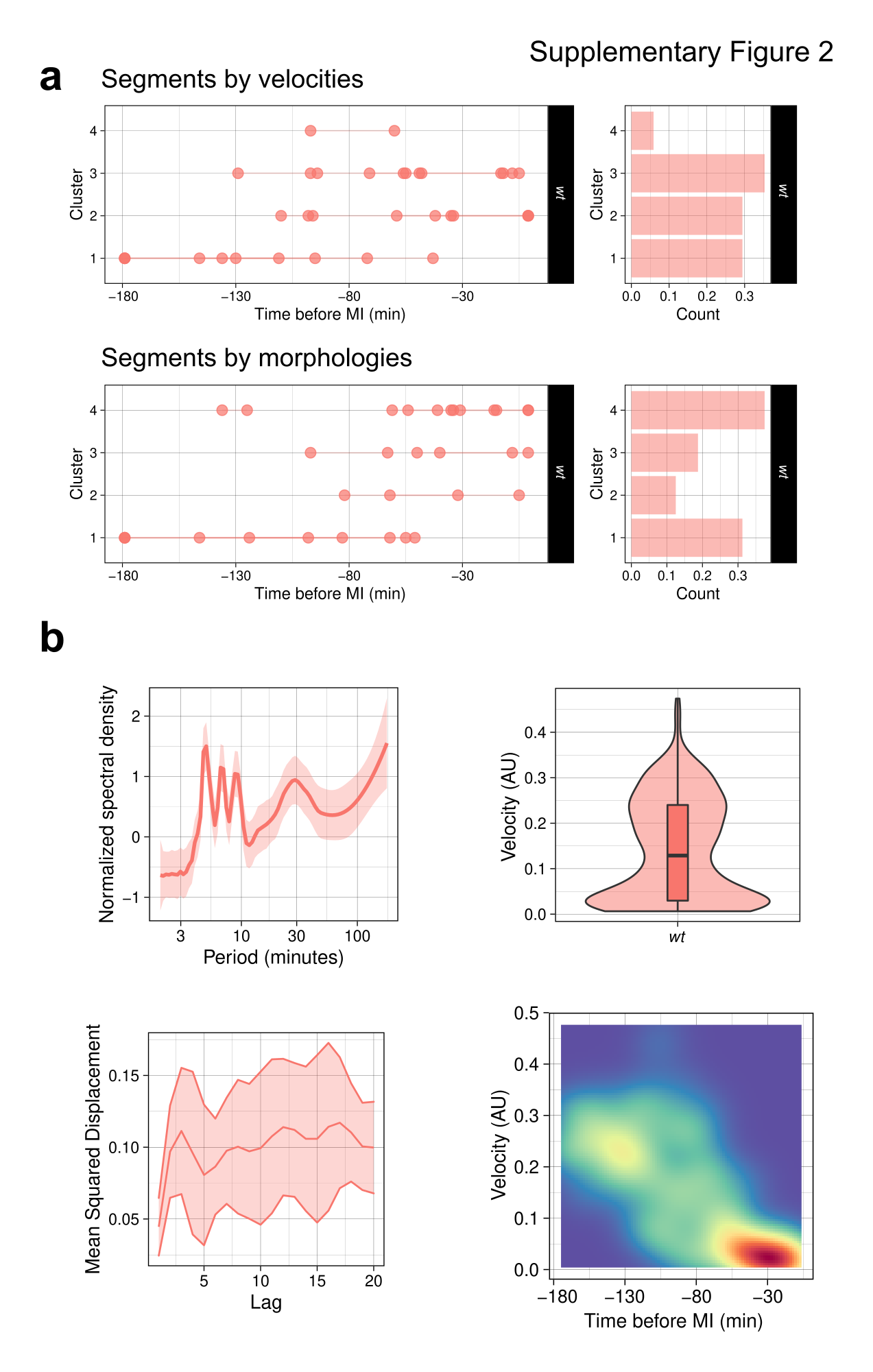

Supplement: Supplementary file 1 [file cells-10-02013-s001.zip › SuppFigure2.tiff]

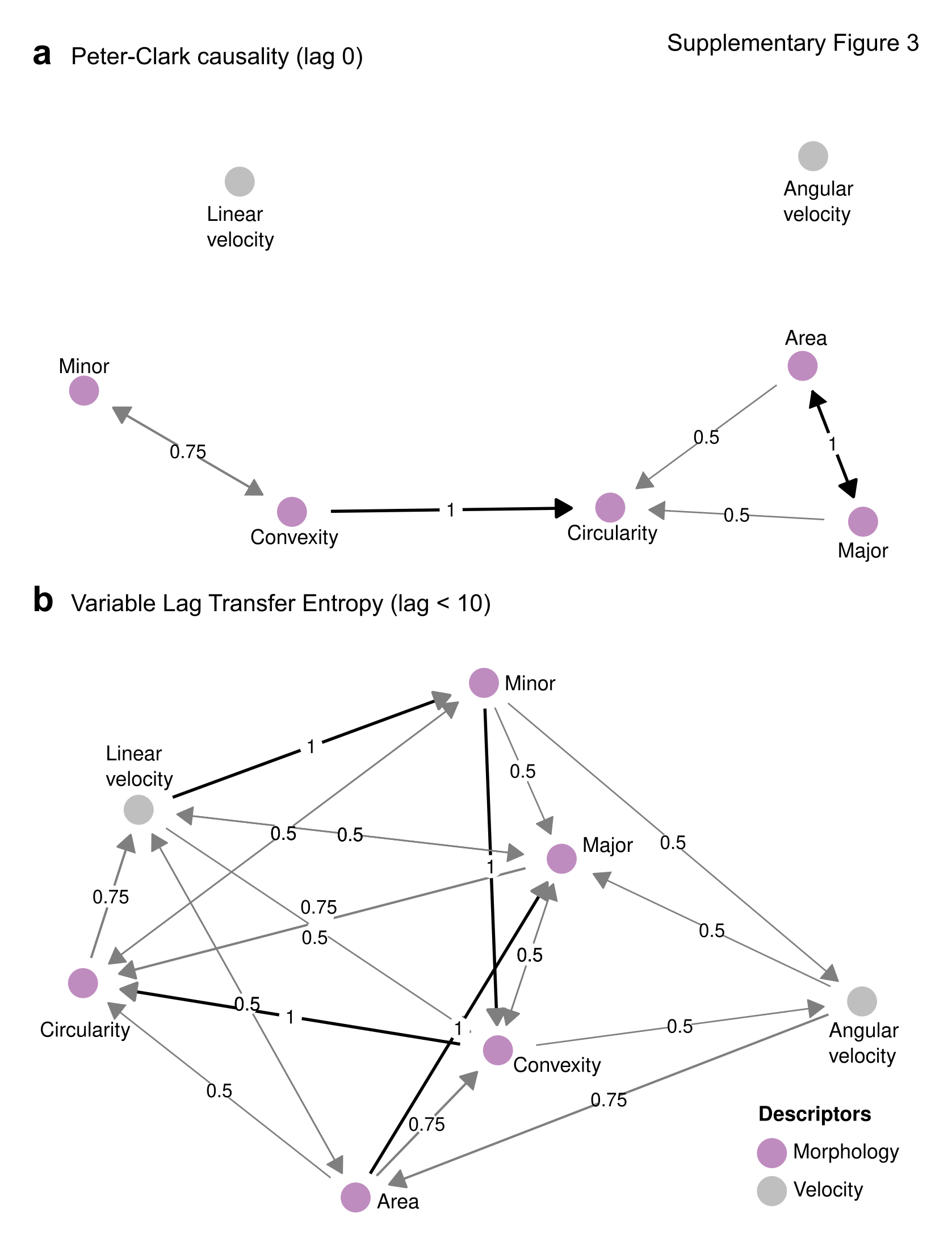

Supplement: Supplementary file 1 [file cells-10-02013-s001.zip › SuppFigure3.tiff]

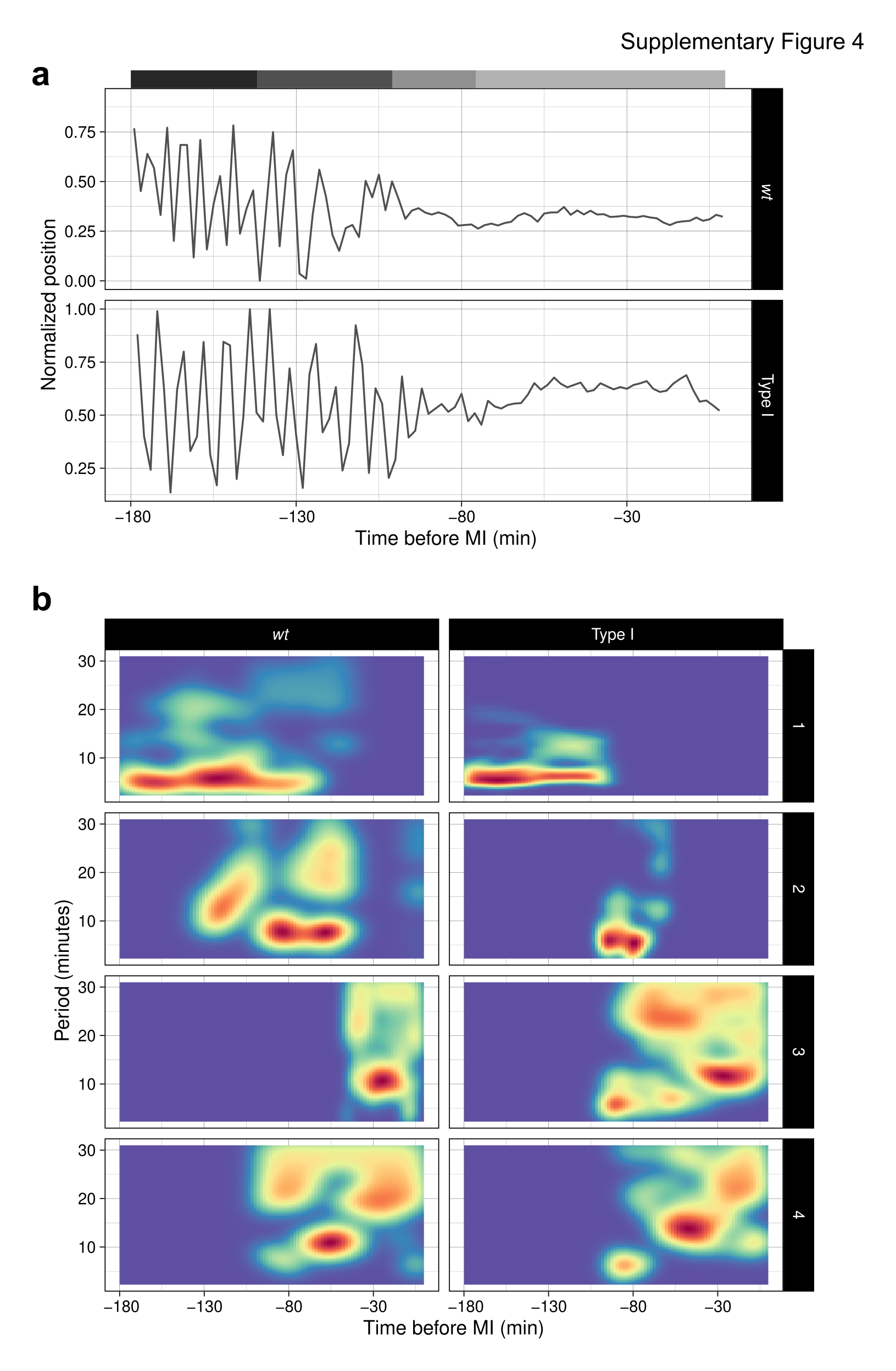

Supplement: Supplementary file 1 [file cells-10-02013-s001.zip › SuppFigure4.tiff]

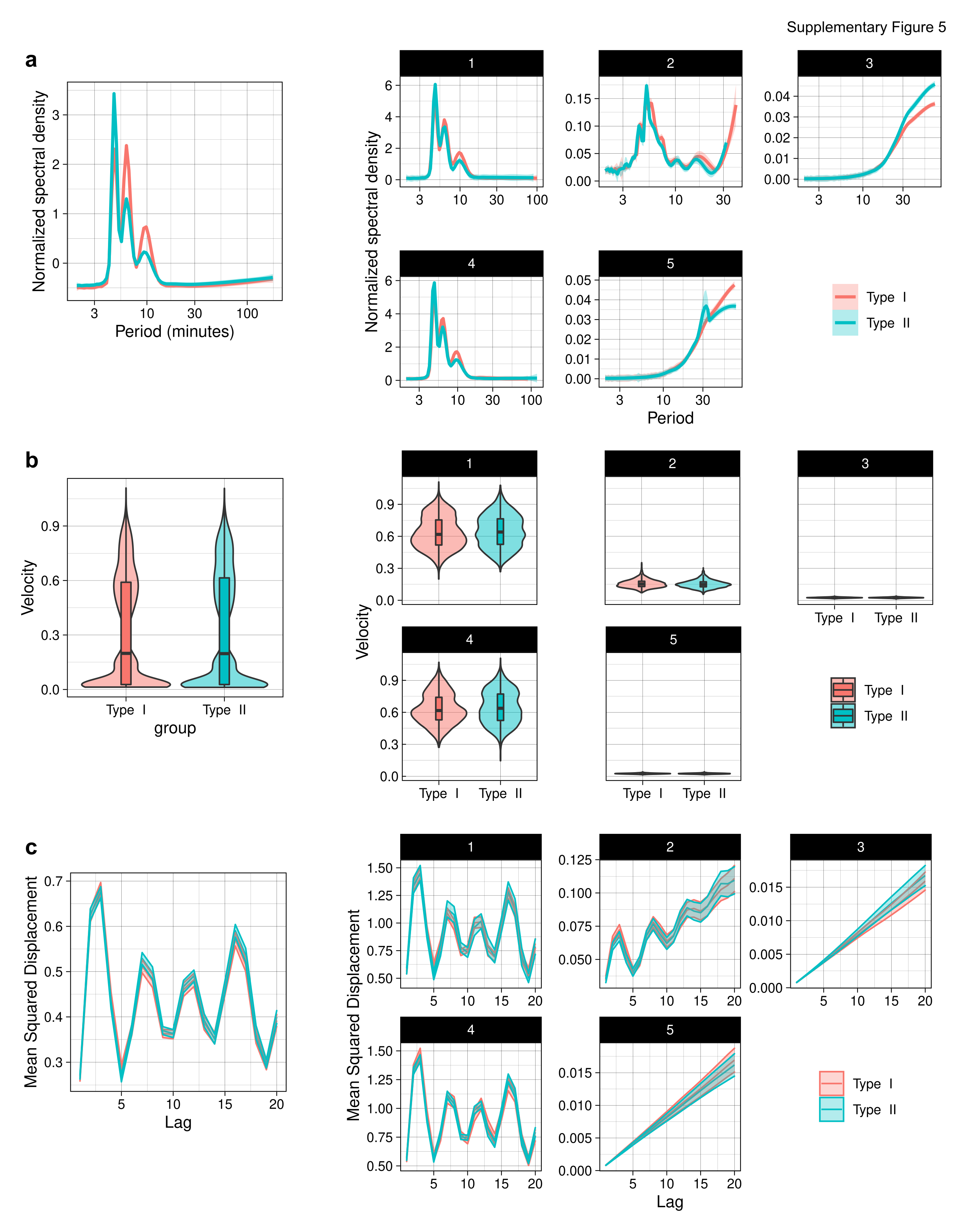

Supplement: Supplementary file 1 [file cells-10-02013-s001.zip › SuppFigure5.tiff]
